# Vitamin A deficiency causes islet dysfunction by inducing islet stellate cell activation via cellular retinol binding protein 1

**DOI:** 10.7150/ijbs.37861

**Published:** 2020-01-30

**Authors:** Yunting Zhou, Junming Zhou, Bo Sun, Wei Xu, Ming Zhong, Yumin Li, Cong He, Yang Chen, Xiaohang Wang, Peter M Jones, Zilin Sun

**Affiliations:** 1 Department of Endocrinology, Zhongda Hospital, Institute of Diabetes, School of Medicine, Southeast University, Nanjing, China; 2 Department of Gastroenterology, Jinling Hospital, Medical School of Nanjing University, Nanjing, China; 3 State Key Laboratory of Bioelectronics, School of Biological Science and Medical Engineering, Southeast University, Nanjing, China; 4 Department of Diabetes, School of Life Course Sciences, King's College London, Guy's Campus, London, UK; 5 Department of Biochemistry and Molecular Biology, School of Medicine, Southeast University, Nanjing, China

**Keywords:** vitamin A, β cell, islet stellate cell, activation, CRBP1

## Abstract

**Background:** Vitamin A (VA) plays an essential role in pancreatic homeostasis. Islet stellate cells (ISCs) are VA-storing cells in pancreatic islets. Herein, we have investigated the effect of VA on glucose homeostasis trough regulation of ISCs function in dietary VA deficiency model mice.

**Methods:** Male C57BL/6 mice were randomly fed a VA-sufficient, a VA-deficient (VAD) or a VAD-rescued diet. Glucose metabolism was assessed by glucose tolerance tests and immunohistochemistry. ISCs activation degree was evaluated by immunofluorescence, quantitative PCR and western blotting in both, retinol-treated cultured ISCs and model mice. Changes in ISCs phenotype and their effect on islets were assessed by lentiviral transduction and enzyme-linked immunosorbent assays in a co-culture system.

**Results:** VAD mice showed irregular shaped islet, glucose intolerance, islet size distribution excursions, and upregulated expression of α-smooth muscle actin (α-SMA, marker of ISCs activation). Reintroduction of dietary VA restored pancreatic VA levels, endocrine hormone profiles, and inhibited ISCs activation. Incubation with retinol increased the expression of VA signaling factors in ISCs, including cellular retinol binding protein 1 (CRBP1). The knockdown of CRBP1 maintained the quiescent ISCs phenotype and reduced the damage of activated ISCs on islet function.

**Conclusions:** VA deficiency reduced islet function by activating ISCs in VAD mice. Restoring ISCs quiescence via CRBP1 inhibition could reverse the impairment of islet function caused by activated ISCs exposure.

## Introduction

Vitamin A (VA), present in different forms of retinoids in the body, is essential for tissue homeostasis and has been implicated in the pathophysiology of various diseases through its roles in cellular immunity, differentiation, and apoptosis 1-3. Clinical evidence indicates that serum VA-related metabolite levels are associated with the incidence of type 2 diabetes 4, 5. VA deficiency during the early stages of pancreatic development decreases β cell mass, which can be attributed to a reduced rate of β cell replication, and results in impaired glucose tolerance in adulthood 6-9. In adult mouse pancreas, a decrease in pancreatic VA level reduced β cell mass increased α cell mass with concomitant hyperglycaemia, and altered serum insulin and glucagon profiles 9-11. These data suggested that VA plays an important role in the maintenance of normal endocrine function. However, the molecular basis of these functions is not well understood.

Islet stellate cells (ISCs) were recently identified by single-cell transcriptome technology as a subset for pancreatic stellate cells (PSCs) in islets 12, 13. ISCs can be divided into two classes based on their phenotype. Quiescent ISCs are enriched in lipid droplets containing retinoid predominantly as retinyl palmitate cytosolic droplets. They express desmin and glial fibrillary acidic protein under physiological conditions, but could be induced by various environmental stimuli to proliferate and generate fibrotic extracellular matrix (ECM), resulting in their transformation from a quiescent cells (resting state) to myofibroblast-like cells (activated state) with concomitant disappearance of the lipid droplets 14. These activated ISCs specifically express α-smooth muscle actin (α-SMA) and secrete collagen I (Col I), collagen III (Col III), fibronectin (FN) and other ECM proteins that would promote islet fibrosis 15. Moreover, we have previously showed that activated ISCs inhibited proliferation and induced apoptosis of INS-1 rat insulinoma cells 16. Therefore, activated ISCs could impair islet endocrine responses and might be involved in islet function.

Retinoid signaling, including retinoic transcription nuclear receptors, intracellular retinoid metabolic enzymes and carrier proteins, affects the composition and the structure of ECM resulting in alteration of organ functions, and pathological consequences in kidney, lung, liver and other organs 17. To date, there have been no studies investigating the biochemical and/or molecular link between VA and ISCs, particularly in islets. To address this issue, the present study examined the effect of VA deficiency on ISCs activation. Our results provide novel insight into the mechanism underlying retinoid-mediated islet homeostasis.

## Materials and Methods

### Animals and diet

Male C57BL/6 six weeks old mice were purchased and maintained in a specific pathogen-free room at the animal research center of University. Model mice were conducted using described methods according to Ref. 18 with some modifications 18. Animals were housed under standard conditions with free access to food and water. Mice were randomly separated into VA-deficient (VAD) diet group (without VA [<120 IU/kg]), and 6- or 12-weeks VA-sufficient (VAS) diet group (15,000 IU/kg VA supplementation) (n = 10-20 per group). After 12 weeks, cohorts of VAD diet group mice (18 weeks old, n = 10) were switched to VAD-rescued (VADR) diet (35,000 IU/kg VA supplementation) for another 8 weeks. All diets were purchased from Animal Diets Co., Ltd (Changzhou, China). The caloric intake details of all groups were matched. At the indicated times, mice were subjected to metabolic testing and sacrificed. The serum and tissue samples were collected and immediately stored at -80°C until further use. All experimental procedures were conducted in accordance with the National Institutes of Health Guide for the Care and Use of Laboratory Animals (NIH Publication No. 8023, revised 1978). More, all animal experiments were approved by the Animal Care and Welfare Committee of Southeast University.

### ISCs isolation and expansion

ISCs were isolated using a previously described method with some modifications 19. Islets obtained from mouse pancreas were digested into single cells with 0.25% trypsin. The cell suspension was centrifuged in 28.7% (w/v) Nycodenz solution at 1400 × *g* for 20 min. Then isolated ISCs were seeded and cultured in Dulbecco's modified Eagle's medium/F12 supplemented with 10% fetal bovine serum and 1% penicillin-streptomycin (all from Gibco, Grand Island, NY, USA). The cells were expanded for 3 to 6 passages before use.

### Islet isolation and co-culture

Mouse islets were obtained according to the standard protocol established in our laboratory 20. Isolated islets were prepared for experiments under the same culture conditions as ISCs. Immediately after ISCs had attached to the culture plate, freshly isolated islets (50 per dish) were placed in the upper chamber. The plate was incubated at 37°C with 5% CO_2_ for the indicated times before analysis.

### Intraperitoneal Glucose Tolerance Test (IPGTT)

For the IPGTT, blood samples were obtained via the tail vein of mice from the experimental and control groups (8-10 mice per group). After fasting for 8 h, mice were given D-glucose (2 g.kg^-1^ of body weight) and the tail vein blood glucose level was measured at 0, 15, 30, 60, 90, and 120 min using a portable glucose monitor (Bayer, Geneva, Switzerland). The area under the curve (AUC) for blood glucose (AUC_IPGTT-glucose_) and serum insulin (AUC_IPGTT-insulin_) were calculated using Sigma Plot software (Systat, San Jose, CA, USA). Fasting blood glucose levels were measured after 8 h fasting. Random blood glucose levels were measured at two or three random time points weekly.

### High Performance Liquid Chromatography (HPLC)

For pancreatic tissue retinol levels measurement, the frozen tissues (about 100-200 mg) were minced into small pieces in ice-cold PBS (phosphate-buffered saline) and rinsed thoroughly for 30 s. After tissue pieces were homogenized (tissue weight (g): PBS (ml) volume=1:1) in glass homogenizers, pancreatic retinoid was extracted by 350 µl of organic solution (acetonitrile/butanol, 50:50, v/v) and collected in the dark for further experiments. Both retinol levels in serum and tissues were detected at a wavelength of 340 nm using a Waters Millennium system (Waters, USA) at Shanghai Adicon Clinical Laboratories. The levels of tissue retinol were normalized to mg of the tissue weight.

### Enzyme-linked immunosorbent assay (ELISA)

Insulin content of serum, cells and cell culture supernatant was measured using an ultrasensitive mouse-specific ELISA kit (MeilianBio, Shanghai, China) according to the manufacturer's instructions.

### Quantitative PCR (q-PCR)

Total RNA was extracted from cells using TRIzol reagent (Life Technologies, Carlsbad, CA, USA) and was reverse transcribed using 5× All-In-One MasterMix (Abcam, Cambridge, MA, USA) on a Real Time PCR iCycler (Thermo Fisher Scientific, Waltham, MA, USA). qPCR was performed using SYBR Green PCR Master Mix (Takara Bio, Otsu, Japan) with gDNA eraser. Mouse-specific primers for target gene amplification (Table 1) were designed based on sequences in the GenBank database. Amplification was performed on a Step One Real-Time PCR System (Applied Biosystems, Foster City, CA, USA) under the following conditions: 95°C for 30 s, followed by 40 cycles of 95°C for 5 s and 60°C for 30 s. Relative mRNA levels were quantified with the ΔCt method with β-actin as the internal reference.

### Western blotting

Proteins were extracted from tissues and cells using radioimmunoprecipitation assay buffer containing protease inhibitor cocktail (Solarbio, Beijing, China). Protein concentration was determined with the bicinchoninic acid assay (KeyGen Biotech, Nanjing, China). Equal amounts of protein (20 μg) were separated by 10% or 8% sodium dodecyl sulphate-polyacrylamide gel electrophoresis and transferred to a polyvinylidene difluoride membrane (Millipore, Billerica, MA, USA) that was blocked with 5% milk and then incubated overnight at 4°C with rabbit antibodies against Col I, FN, and α-SMA (all from Abcam) or mouse antibodies against CRBP1 (Santa Cruz Biotechnology, Santa Cruz, CA, USA) or β-actin (ZSGB-Bio, Beijing, China) used at 1:1000 dilution. Horseradish peroxidase-conjugated goat anti-rabbit or -mouse secondary antibody (KeyGen Biotech; 1:3000 dilution) was applied for 1 h at room temperature. Protein bands were detected by enhanced chemiluminescence (Millipore) using a Fluor Chem-FC2 imaging system (Alpha Innotech, San Leandro, CA, USA), and signal intensity was quantified using Image J software.

### Histology, immunohistochemistry and immunofluorescence

Paraffin-embedded pancreas and liver tissue sections (4 µm) were stained with hematoxylin and eosin (H&E) for histological examination. Sections were fixed with 4% formaldehyde, permeabilised with 0.1% Triton X-100 in PBS, blocked with 5% bovine serum albumin, and then incubated with rabbit anti-glucagon/α-SMA/Col I/FN antibody (Cell Signaling Technology, Danvers, MA, USA; 1:200 dilution) and mouse anti-insulin/C-peptide antibody (all from Abcam; 1:200 dilution). For immunohistochemistry, sections were incubated with the HRP-conjugated anti-rabbit secondary antibody (ZSGB-BIO, China) and developed with the HRP- Diaminobenzidine kit (DAKO, Copenhagen, Denmark). For double-labelling immunofluorescence, we used Alexa Fluor 488- or 568-conjugated secondary antibodies from different host species (Abcam; 1:200 dilution). The cells were stained with the nuclear dye DAPI (Invitrogen, Carlsbad, CA, USA) and then examined on an inverted microscope (Nikon, Tokyo, Japan). For the islet morphology analysis, it was performed as described previously with some modification 21-23. All sections from each group, between 250 and 300 immunopositive islet fields, were determined. Selection criteria included clear presence of the nucleus within islets, the ability to clearly visualize nuclear boundaries, circular shape (similar dimensions in all directions), and the appearance to the observer that the nucleus had been sectioned through its maximum diameter. Islet morphology that includes unstained fractions such as intraislet capillaries is measured by automated contouring of each islet structure. Each section was analyzed separately using Image Pro Plus 4.5.1 software (Media Cybernetics, Silver Springs, MD).

### Lentiviral transfection

Lentiviruses for CRBP1 overexpression and interfering were obtained from GenePharma (Shanghai, China). ISCs from passages 3 to 6 were infected with CRBP1 overexpression/interfering vector/a negative control (green fluorescent protein vector), at a multiplicity of infection of 10 in the presence of 5 μg.mL^-1^ polybrene as previously described 24. Stable clones were selected by supplementing the medium with 2 μg.mL^-1^ puromycin for 6 days. The transfection efficiency was confirmed by western blotting and qPCR analysis.

### Cell viability and apoptosis assays

Cell viability was evaluated with a colorimetric MTS assay kit (Promega, Madison, WI, USA) according to the manufacturer's instructions, with absorbance measured at 490 nm. For terminal deoxynucleotidyl transferase-mediated dUTP-biotin nick end labeling (TUNEL) assay, islet DNA fragmentation was evaluated in paraffin-embedded sections using DNA fragmentation detection kit (Roche, Germany) according to the manufacturer's protocol.

### Cell migration and proliferation assays

For the transwell migration assay, 3×10^4^ ISCs were serum-starved for 24 h in 24-transwell plates using inserts with a pore size of 8 μm (Millipore, USA). The cells were then incubated for 24 h at 37°C, fixed with 4% formaldehyde, and stained with 1% crystal violet for 10 min. For the wound-healing assay, 3×10^5^ cells were seeded in 6-well culture plates and grown to reach confluence. The monolayers were wounded by scraping off a strip of cells with a 20 μl pipette tip. After 24 h, cells migrating into the wound boundaries were counted manually. Cells migration was visualized by light microscopy and the area was calculated using Image J software.

For CCK-8 assay, Cells were suspended at a final concentration of 2×10^3^/well and cultured in 96-well microplates for 8 h, 24 h, and 48 h, after which, CCK-8 reagent (10 μl, Keygen, Biotech) was added to each well containing 100 µl of culture medium and the plate was incubated for 1 h at 37°C. Viable cells were evaluated by measuring the absorbance of each well using an auto microplate reader (BioTek Instruments, Inc, USA).

### Statistical analysis

Data are representative of at least three independent experiments and are expressed as mean ± standard error. Differences between two groups and among more than two groups were evaluated with the Student's t test and analysis of variance (ANOVA), respectively. Standard regression analysis was used to evaluate linear relationships between pancreatic islet area and percentage of α-SMA expression in islets. All statistical analyses were performed using Prism v.6.0 software (GraphPad, La Jolla, CA, USA). Significance was assumed at P < 0.05.

## Results

### The glucose metabolic phenotypes of mice were damaged in different degree of VA deficiency

**Figure 1:**
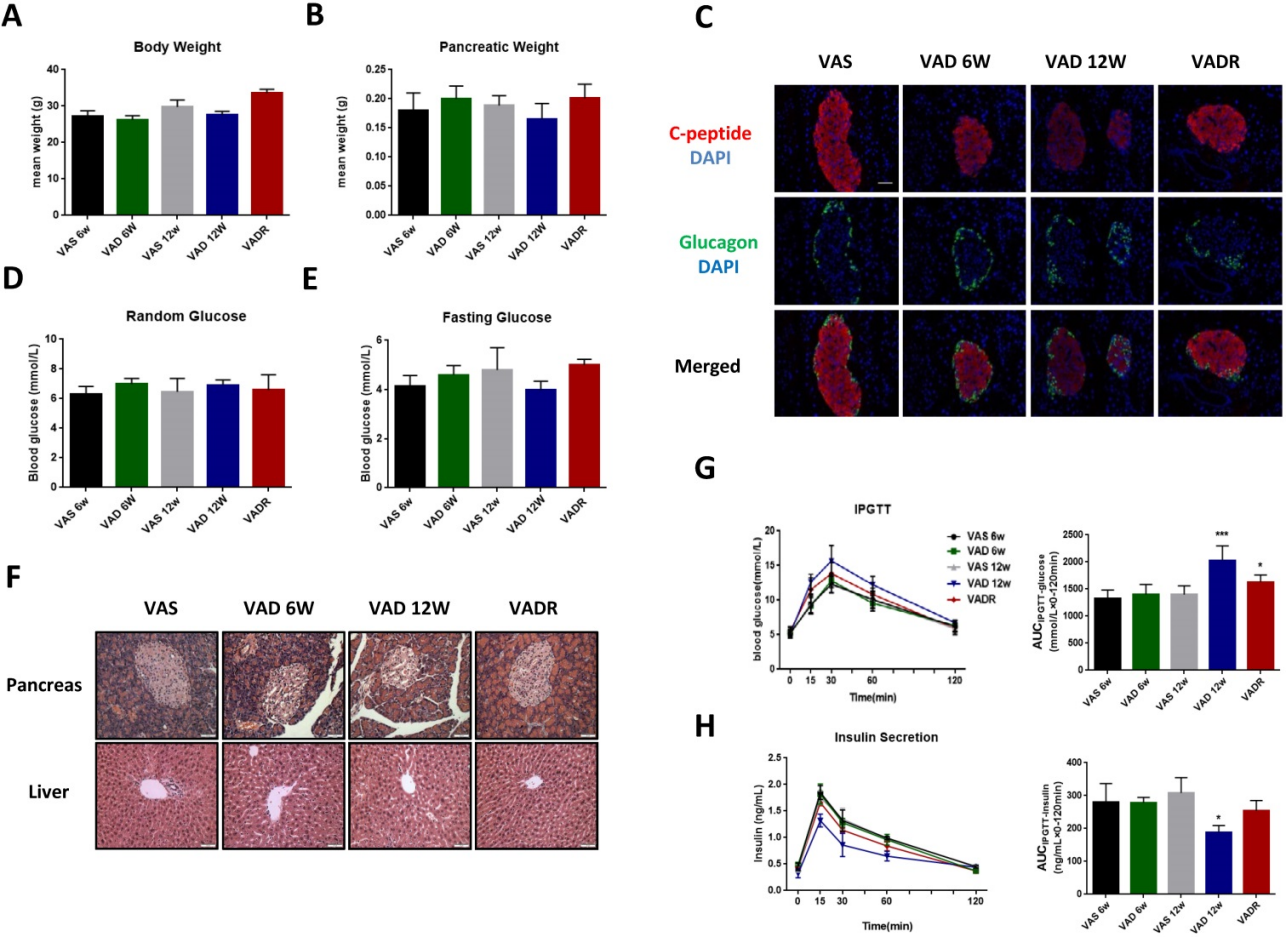
** The glucose metabolic phenotypes of mice in different degree of VA deficiency.** (A, B) Body and pancreatic weights of VAS, VAD 6w, VAD 12w and VADR mice. (C) Representative photomicrographs of C-peptide and glucagon double-stained pancreatic islets from VAS, VAD 6w, VAD 12w, and VADR mice. (D, E) Random and fasting glucose levels of VAS, VAD 6w, VAD 12w and VADR mice. (F) Representative photomicrographs of H&E stained pancreatic and liver sections from VAS, VAD 6w, VAD 12w, and VADR mice. (G, H) Islet function of VAS, VAD 6w, VAD 12w and VADR mice was analyzed via the IPGTT (i.e., AUC_IPGTT-glucose_ and AUC_IPGTT-insulin_). Magnification, 40×; Scale bars, 50 μm. * = P < 0.05, *** = P < 0.001 in post-hoc comparisons between each VAD group and the control group after one-way ANOVA analysis, showing a significant effect.

After 6 and 12 weeks of VA deprivation (VAD 6w and VAD 12w), VAD 12w mice tended to have lower body and pancreas weights than control mice, nevertheless, no significant difference was found (Fig 1A, B). The production of local islet hormones was examined by immunofluorescence labelling, and we found significant reductions of C-peptide signal in VAD 6w and VAD 12w mice compared to VAS mice (Fig 1C). Random and fasting glucose levels did not differ between VAD and VAS mice (Fig 1D, E). Pancreatic VA levels in VAD 6w and VAD 12w mice were 32% and 63% lower, respectively, than these of VAS mice. Meanwhile, there were no significant changes in serum VA levels among these groups (Table S1). Histological analysis of pancreatic islets by H&E staining revealed that severe VA deficiency caused abnormal disorder of islet cells with non-circular shaped boundaries, which is more obvious in VAD 12w mice (Fig 1F). In the glucose tolerance test, as shown in Fig 1G, H, a slight non-significant increment of AUC_IPGTT-glucose_ and a reduction of AUC_IPGTT-insulin_ in VAD 6w mice was observed compared to VAS mice; interestingly, the same tendency was more evident over time and was statistically significant in VAD 12w mice.

### VA deficiency causes changes in islet size distribution and ISCs activation

**Figure 2:**
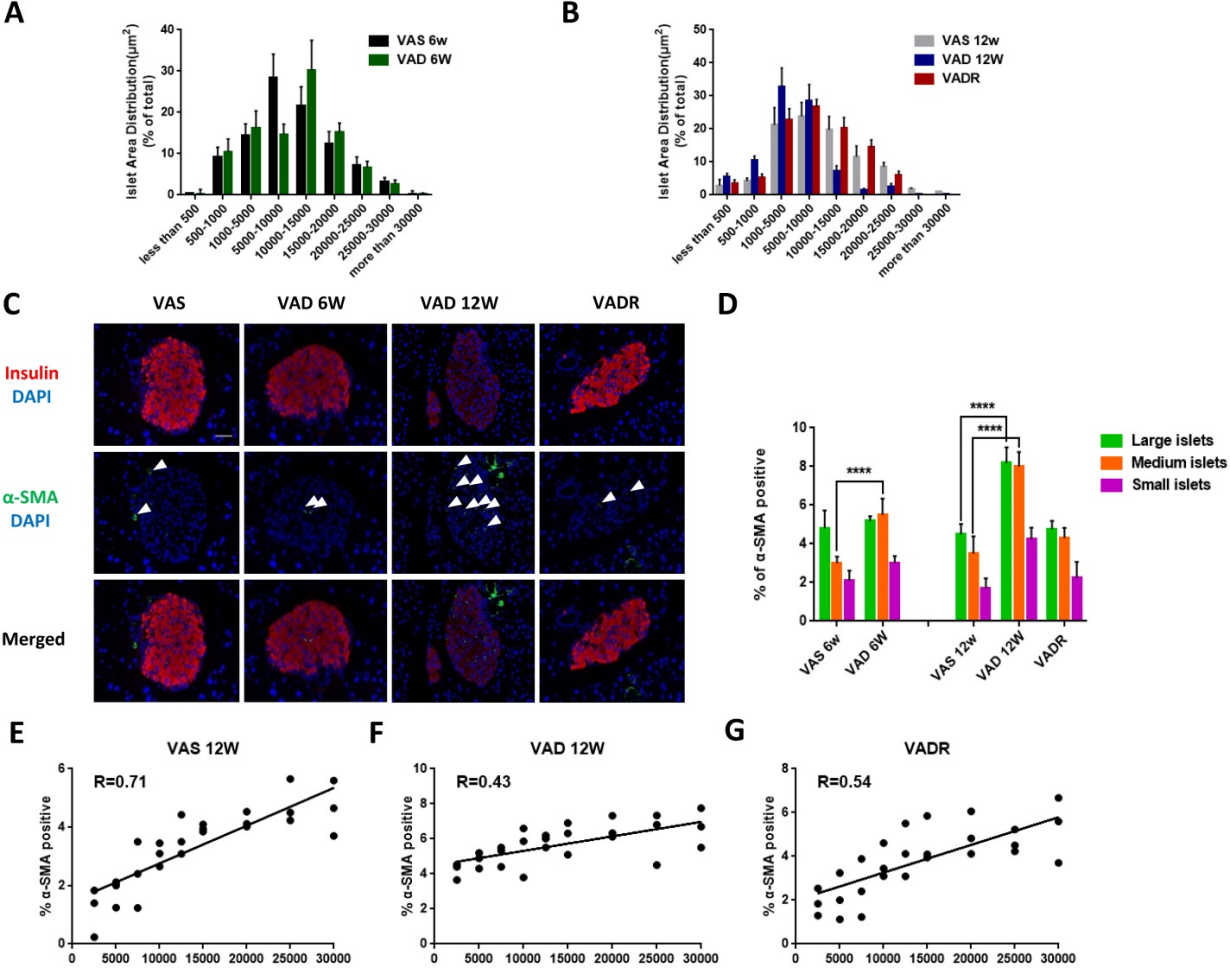
** Dietary VA deprivation causes islet size distribution excursions and ISCs activation.** (A, B) Relative percentages of different size islets were quantified in VAS, VAD 6w, VAD 12w, and VADR mice. (C) Representative photomicrographs of activated ISCs and β cells were double immunofluorescence stained with antibodies against α-SMA and insulin. (D) Percentages of α-SMA fluorescent signals were measured and quantified of large islets, medium islets, and small islets. (E-G) Correlation analysis of percentages of α-SMA-positive ISCs and islets size distribution in VAS, VAD 6w, VAD 12w, and VADR mice. Magnification, 40×; Scale bars, 50 μm. **** = P < 0.0001 in post-hoc comparisons vs control group after two-way ANOVA analysis. Linear relationships between pancreatic islet size distribution and percentages of α-SMA expression in ISCs were determined by regression analysis.

In terms of pancreatic islet size distribution, VAD 6w mice showed a 10% reduction in the number of large-sized islets (> 20,000 μm^2^) but no differences in medium-sized (5000-20,000 μm^2^) or small-sized (< 5000 μm^2^) islets were observed compared to VAS mice (Fig 2A). However, we observed additional changes in islet size distribution of VAD 12w mice, including a 70%, 32%, and 72% fewer large-, medium-, and small-sized islets, respectively (Fig 2B). We used double immunofluorescence labelling to determine whether VA deficiency increases α-SMA expression in islets. Insulin immunoreactivity of the islets decreased in VAD mice, while was accompanied by upregulation of α-SMA, Col I, and FN indicating ISCs activation. This phenotype was reversed in VADR mice (Fig 2C). More, an increase of α-SMA expression in PSCs rather than that in hepatic stellate cells (HSCs) of VAD mice was detected (Fig S1A). Nevertheless, the expression of Col I and FN increased in both ISCs and PSCs of VAD mice compared to VAS mice (Fig S1B). VA deficiency did also induce detectable apoptotic cells in islets with DNA strand breakage. By week 8 of VA diet rescue, VADR mice displayed greatly diminished cell apoptosis in islets compared to VAD mice (Fig S1C). Given the larger fraction of α-SMA-positive ISCs in the islet of VAD mice, we investigated whether α-SMA is differentially expressed in large-, medium-, and small-sized islets (Fig 2D). The results of the regression analysis showed that the percentage of α-SMA-positive ISCs was in correlation with islet size in VAS 12w mice (R = 0.71, Fig 2E), while a weaker correlation was observed in VAD 12w mice (R = 0.43, Fig 2F). Dietary VA supplementation for 8 weeks restored the positive relationship between α-SMA expression and islet size in VADR mice (R = 0.54, Fig 2G).

### Retinol inhibits ISCs activation *in vitro*

**Figure 3:**
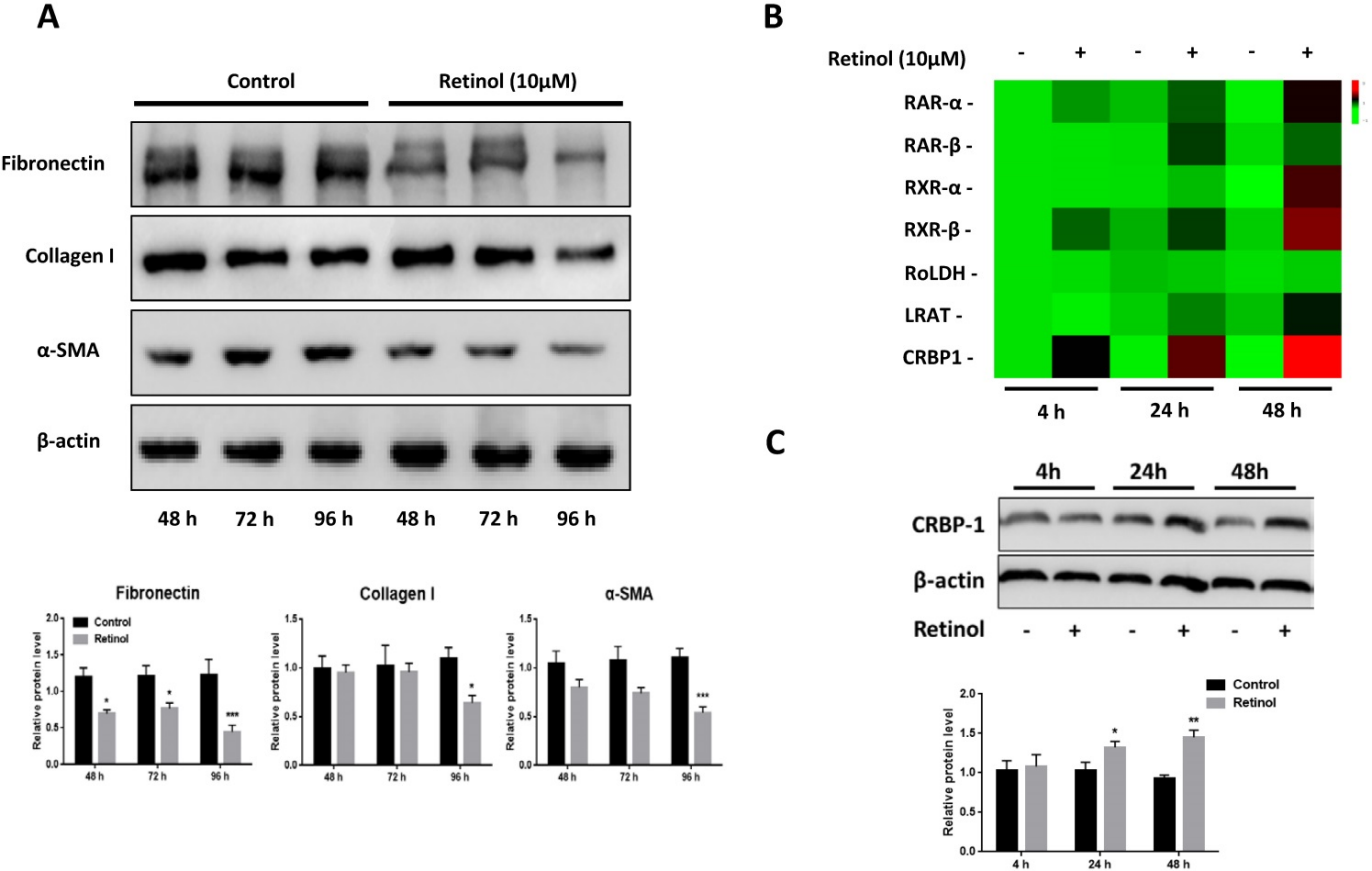
** Retinol inhibits ISCs activation.** (A) The protein levels of α-SMA, Col I, and FN in ISCs were analyzed by western blotting with retinol treatment for 48 h, 72 h, and 96 h. (B) The mRNA levels of VA metabolic molecules were analyzed by qPCR with retinol treatment 4 h, 24 h, and 48 h. (C) The CRBP1 protein level in ISCs was analyzed by western blotting with retinol treatment for 4 h, 24 h, and 48 h. * = P < 0.05, ** = P < 0.01, *** = P < 0.001 in post-hoc comparisons vs control group after one-way ANOVA analysis, showing a significant effect.

After 48 h incubation of ISCs with retinol (10 μM), a reduction of α-SMA, FN and Col I expressions was observed by western blotting, and this persisted after 72 and 96 h (Fig 3A). To investigate the role of VA-associated molecules in ISCs activation, the mRNA levels of retinoic acid receptors α (RARα), retinoic acid receptors β (RARβ), retinoid X receptors α (RXRα), retinoid X receptors β (RARβ), retinol dehydrogenase (RoLDH), lecithin retinol acyltransferase (LRAT), and cellular retinol binding protein (CRBP1) increased in a time-dependent manner relative to the control group, with CRBP1 being the highest expressed (Fig 3B). The same pattern was observed for protein levels (Fig 3C).

### CRBP1 alters the phenotype of activated ISCs and affects islets survival and function

**Figure 4:**
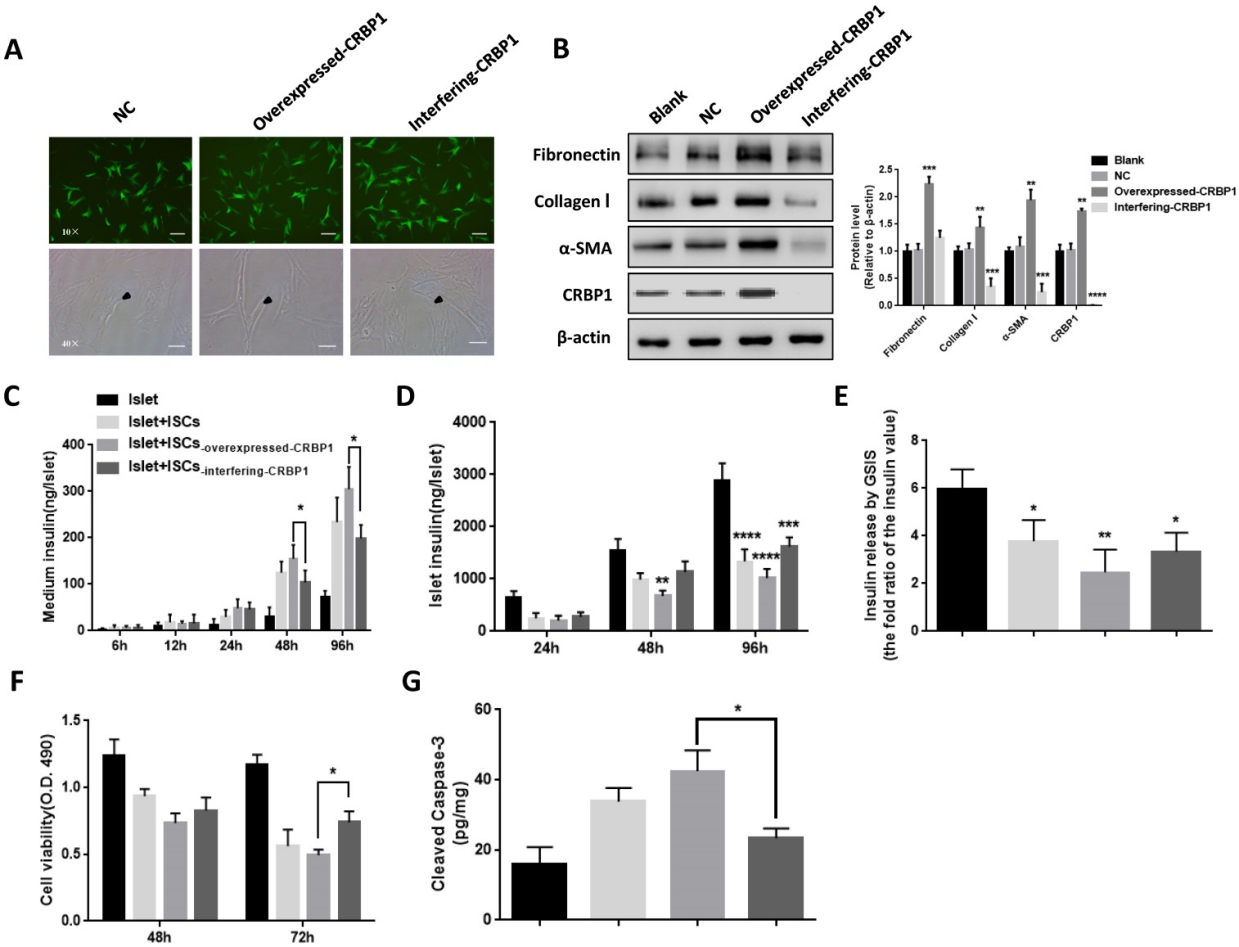
** CRBP1 reverses ISCs phenotype and restores islet function.** (A) Representative photomicrographs of ISCs transduced with CRBP1 gene overexpressed/interfering/GFP (NC)**.** The phenotype change of activated ISCs was observed and indicated by black arrows. (B) The protein expression of α-SMA, Col I, and FN in ISCs_-interfering-CRBP1_, ISCs_-overexpressed-CRBP1_, ISCs_-NC_, and ISCs_-blank_ was detected by western blotting. (C-F) Islet function/survival was measured at indicated time points in co-culture system. Magnification, 10×, 40×; Scale bars, 100 μm, 50 μm, respectively. * = P < 0.05, ** = P < 0.01, *** = P < 0.001, **** = P < 0.0001 in post-hoc comparisons vs control group after one-way or two-way ANOVA analysis, showing a significant effect.

ISCs_-interfering CRBP1_ had the classical polygonal appearance similar to the one of quiescent ISCs and a reduced protein expression of α-SMA, FN, and Col I compared with ISCs_-NC_ (Fig 4A, B). ISCs_-overexpressed CRBP1_ displayed a higher migration and proliferation rate compared to ISCs_-NC_, while the opposite trend was detected in ISCs_-interfering CRBP1_ (Fig S2A, C). We further investigated whether the addition of retinol could re-inhibit the activated state of ISCs_-overexpressed CRBP1_. After incubation with retinol for 96 h, the α-SMA expression in ISCs_-overexpressed CRBP1_ did not significantly decrease relative to that in ISCs_-NC_ (Fig S2B). These results indicate that CRBP1 is the key mediator for VA transcriptional regulation to maintain the quiescent ISCs phenotype.

In co-cultured with ISCs_-overexpressed CRBP1_ at 48 h, the insulin level in the supernatant was 24% higher compared to ISCs_-NC_. The insulin level continued to rise, reaching a 1.3-fold at 96 h. However, in co-cultured with ISCs_-interfering CRBP1_, the insulin level was reduced by 16% at 48 h (Fig 4C). Similarly, islets co-culture with ISCs_-NC_ exhibited a progressive increase in cellular insulin content. By comparison, islets insulin content in co-cultures with ISCs_-interfering CRBP1_ and ISCs_-overexpressed CRBP1_ showed 1.2-fold elevation and 25% reduction, respectively, compared to ISCs_-NC_ at 96 h (Fig 4D). After co-culturing with ISCs_-interfering CRBP1_ for 72 h, the insulin release ability and islets cell viability improved by 35% and 48%, respectively, compared with those with ISCs_-overexpressed CRBP1_ (Fig 4E,F). Likewise, the augmentation of the concentration of cleaved caspase-3 in islets induced by CRBP1-overexpressed exposure were significantly reduced in CRBP1 interfering exposure at the same time point (Fig 4G). Thus, CRBP1 plays a critical role in maintaining ISCs phenotype and consequently, islets survival and function.

## Discussion

VA deficiency contributes significantly to the global burden of disease, particularly affecting resource-constrained countries. In our present study, we have used VA-sufficient diet-fed mice as control group. For the VADR group, the purified diet for rodents contained excess levels of VA, so the recovery of VAD diet-fed mice is due to VA supplementation. Our results showed that decreased VA level in the pancreas due to the prolonged VAD diet has induced changes in islet morphology and altered glucose metabolism profile. Notably, other studies have also reported that VA deprivation increased glucose excursions, decreased pancreatic insulin content, and induced apoptosis in pancreatic islets 18, 25, 26. We confirmed that VA deficiency altered islets size distribution and damaged islet function. VADR treatment abolished most of these effects, thereby restoring the glucose metabolism phenotype to that observed in mice on a VAS diet. Therefore, VA is required to maintain normal islets function.

Our study showed that the percentage of α-SMA-positive cells increased in VAD mice as compared to VAS mice, whereas VADR mice showed opposite trend suggesting that VA could restore and preserve the quiescent state of ISCs 27-29. Thus, prolonged VA deficiency alters the phenotype of resting ISCs to that of myofibroblast-like cells, leading to increased ISCs activation. Given that stellate cells are found in numerous tissue types, including the exocrine pancreas and liver, we also performed immunohistochemistry staining of α-SMA to determine the effects of VA deprivation on the other major stellate cell populations. We have detected an increase in α-SMA expression in PSCs instead of HSCs in VAD mice (Fig S1A). Some studies showed that retinol value in liver and pancreas was 51.24 pmol/mg and 1.54 pmol/mg in normal mice, respectively 30. Quiescent HSCs are responsible for storing up to 80% of hepatic retinoid and can respond to the VA needs of target tissues 31, 32. Since retinoid is under tight hepatic homeostatic control, and the pancreas has few retinoid-storing cells compared with liver 33, the increase in α-SMA expression provides pancreatic cells an early cue of diminishing VA availability and, if VA levels continue to drop, the liver retinol storage would be reduced leading to an intrinsic cascade of HSCs activation. In pervious study, our group has compared the biological characteristics of ISCs with typical PSCs, and found that there are 32 significant differentially expressed genes between activated PSCs and activated ISCs. Due to fewer lipid droplets, ISCs appeared to be more easily activated by stimulators, and demonstrated reduced proliferation and migration abilities 34. Therefore, the biological phenotypes of ISCs and PSCs are similar, but not completely identical. Considering the similarities in developmental biology and anatomy, we believed that the type of PSCs associated with islet dysfunction may be classified as ISCs, because activated ISCs were conducive to islet fibrosis 16, 35. Based on the reduction in islets size, and increase and decrease in the number of small- and large-sized islet clusters, respectively, we examined the correlation between changes in islets size distributions and the fraction of α-SMA-positive ISCs. We found that α-SMA expression in islets was positively correlated with islets size in VAS mice whereas in VAD mice, the correlation was weaker, although this was restored by dietary VA rescue for 8 weeks. These results suggested that one mechanism by which VA controls islets size is by regulating the size of the activated, α-SMA-positive ISCs pool.

Peroxisome proliferator-activated receptor-γ and sterol regulatory element-binding protein-1c are nuclear receptors that are critical for HSC activation and their ectopic expression induces the transformation of activated to quiescent HSCs 36, 37. This study provides novel evidence that VA induces quiescence in activated ISCs by blocking α-SMA expression and collagen synthesis, as reported in PSCs 27-29. Notably, we found that the above effects were associated with increase in the levels of functional retinoic active metabolites, especially CRBP1, which is required for intracellular delivery of retinol to catalyze the conversion of retinol to retinyl-esters, and to enzymes which metabolize retinol to retinoic acid; hence, it is essential for normal VA metabolic homeostasis and signalling 38-40. Several studies have suggested a correlation between CRBP1 expression and the appearance of α-SMA in activated hepatic fibroblastic cells involved in fibrogenesis 41, 42. CRBP-1 interfering restored the polygonal appearance of quiescent ISCs and reduced the expression of activation-related proteins such as α-SMA, FN, and Col I, producing a resting-state phenotype. Thus, regulation of VA metabolism-related molecules is required to maintain a quiescent ISCs population. Activated ISCs directly impact pancreatic islets cell viability and proliferation 16 and impair islets endocrine functions, leading to islet fibrosis and exocrine pancreatitis. It was reported that activated ISCs respond to the diabetic environment by increasing the activation potential, thereby reducing normal β cell function and/or survival 43. Our data suggest that exposure to ISCs, in which the quiescent phenotype has been restored by CRBP1 interfering, prevents sustained insulin release, which improves intracellular insulin content and islet viability. This may be due to reduced proliferative capacity exhibited by quiescent ISCs, collagen synthesis, and production of proinflammatory cytokines 15, 44. Moreover, these “re-quiescent” ISCs showed enhanced glucose-induced insulin release and basal insulin secretion. Thus, reversing ISCs activation and maintaining their quiescent state, are critical for preserving the function of pancreatic endocrine cells 29. Our observations from the co-culture experiment indicated that ISCs with CRBP1 interfering improved the long-term survival of islets, suggesting that cell-based strategies that blocking ISCs activation could be effective for diabetes treatment. However, additional studies are needed to clarify the mechanisms that maintain the balance between ISCs and β cells.

In summary, two key novel findings were proposed in this study: 1) VA deficiency induced pancreatic islets dysfunction by activating ISCs population. 2) CRBP1 played an important role in ISCs activation under VA deficiency condition.

## Supplementary Material

Supplementary figures and tables.

## Figures and Tables

**Table 1:** Sequences of primers used for q-PCR

Gene	Primer sequence(5'-3')	
RARα	F:CCATGTACGAGAGTGTGGAAGTC	R: CCTGGTGCGCTTTGCGA
RARβ	F: ATCCTGGATTTCTACACCG	R:CACTGACGCCATAGTGGTA
RXRα	F:CGCTCCTCAGGCAAACACTA	R:GGAGGATGCCGTCTTTCACA
RXRβ	F:CTTCGGGAGAAGGTGTACGC	R:GGCAACACTTAGCAGGGTTC
LRAT	F:GCCTCCAAGACTGTCACGAA	R:AGTACAAGCTGGCCTTCGAC
RolDH	F:GCAAAGACTCGTCAGACCCA	R:GATCTCCTCCTGCATCACCG
CRBP1	F:GCTGAGCACTTTTCGGAACT	R:GGAGTTTGTCACCATCCCAG
β-actin	F:AGGGAAATCGTGCGTGACAT	R:CGCAGCTCAGTAACAGTCCG

## Abbreviations

VA: Vitamin A
VAD: VA-deficient
VAS: VA-sufficient
VADR: VAD-rescued
ISCs: islet stellate cells
PSCs: pancreatic stellate cells
HSCs: hepatic stellate cells
ECM: extracellular matrix
α-SMA: α-smooth muscle actin
Col I: collagen I
Col III: collagen III
FN: fibronectin
RARα: retinoic acid receptor α
RAR β: retinoic acid receptor β
RXRα: retinoid X receptors α
RXRβ: retinoid X receptors β
RoLDH: retinol dehydrogenase
LRAT: lecithin retinol acyltransferase
CRBP1: cellular retinol binding protein
IPGTT: intraperitoneal glucose tolerance test
