# Supplementary material for: Vitamin A deficiency causes islet dysfunction by inducing islet stellate cell activation via cellular retinol binding protein 1
Source: Int J Biol Sci. 2020 Jan 30;16(6):947–56. doi: 10.7150/ijbs.37861 (PMC7053333; doi:10.7150/ijbs.37861)
Supplement: Supplementary file 1 — Supplementary figures and tables. [file ijbsv16p0947s1.zip › Supplementary materials/Figure legend.docx]

**Supplementary material (Figure legend):**

**Fig S1, Dietary VA deprivation leads to stellate cells activation and islet cells apoptosis.** (A) The α-SMA expression was detected by immunohistochemistry in pancreatic and hepatic sections from VAS, VAD 6w, VAD 12w, and VADR mice. (B) The Col I and FN expression was detected by immunohistochemistry in pancreatic sections from VAS, VAD 6w, VAD 12w, and VADR mice. (C) Islet sections stained with TUNEL assay from VAS, VAD 6w, VAD 12w, and VADR mice*.* The changes were indicated by black arrows. Magnification, 40×; Scale bars, 50 μm. ** = P < 0.01, *** = P < 0.001 in post-hoc comparisons vs control group after two-way ANOVA analysis, showing a significant effect.

**Fig S2, CRBP1 is the key mediator to maintain the quiescent ISCs phenotype.** (A) Migration abilities of ISCs_-interfering-CRBP1_, ISCs_-overexpressed-CRBP1_, and ISCs_-NC_ was detected by wound healing and transwell assay. (B) The α-SMA, Col I, and FN expression in ISCs_-interfering-CRBP1_, ISCs_-overexpressed-CRBP1_, and ISCs_-NC_ was detected by western blotting with retinol treatment for 48 h and 96 h. (C) Proliferation abilities of ISCs_-interfering-CRBP1_, ISCs_-overexpressed-CRBP1_, and ISCs_-NC_ was detected by CCK-8 assay. Magnification, 10×, 40×; Scale bars, 100 μm, 50 μm, respectively. * = P < 0.05, ** = P < 0.01, *** = P < 0.001in post-hoc comparisons vs control group after one-way or two-way ANOVA analysis, showing a significant effect.
