# Supplementary material for: Vitamin A deficiency causes islet dysfunction by inducing islet stellate cell activation via cellular retinol binding protein 1
Source: Int J Biol Sci. 2020 Jan 30;16(6):947–56. doi: 10.7150/ijbs.37861 (PMC7053333; doi:10.7150/ijbs.37861)
Supplement: Supplementary file 1 — Supplementary figures and tables. [file ijbsv16p0947s1.zip › Supplementary materials/Supplementary materials-FigureS1-2.pptx]

## Slide 1
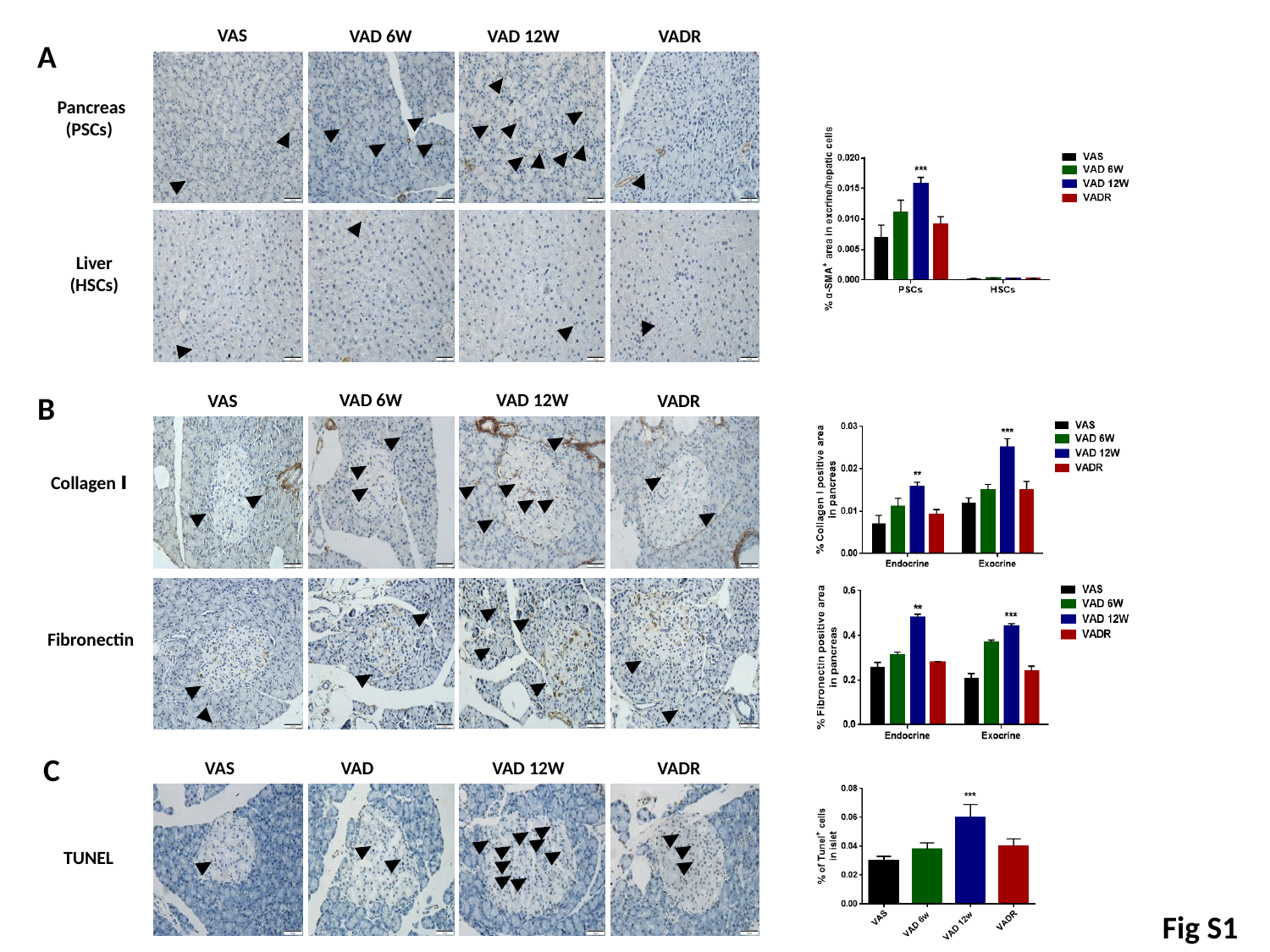

VAS
VAD 6W
VAD 12W
VADR
Pancreas
 (PSCs)
Liver
(HSCs)
A
VAD 12W
B
VAD 6W
VAS
VADR
Collagen Ⅰ
Fibronectin
C
VAS
VAD 6W
VAD 12W
VADR
TUNEL
Fig S1

## Slide 2
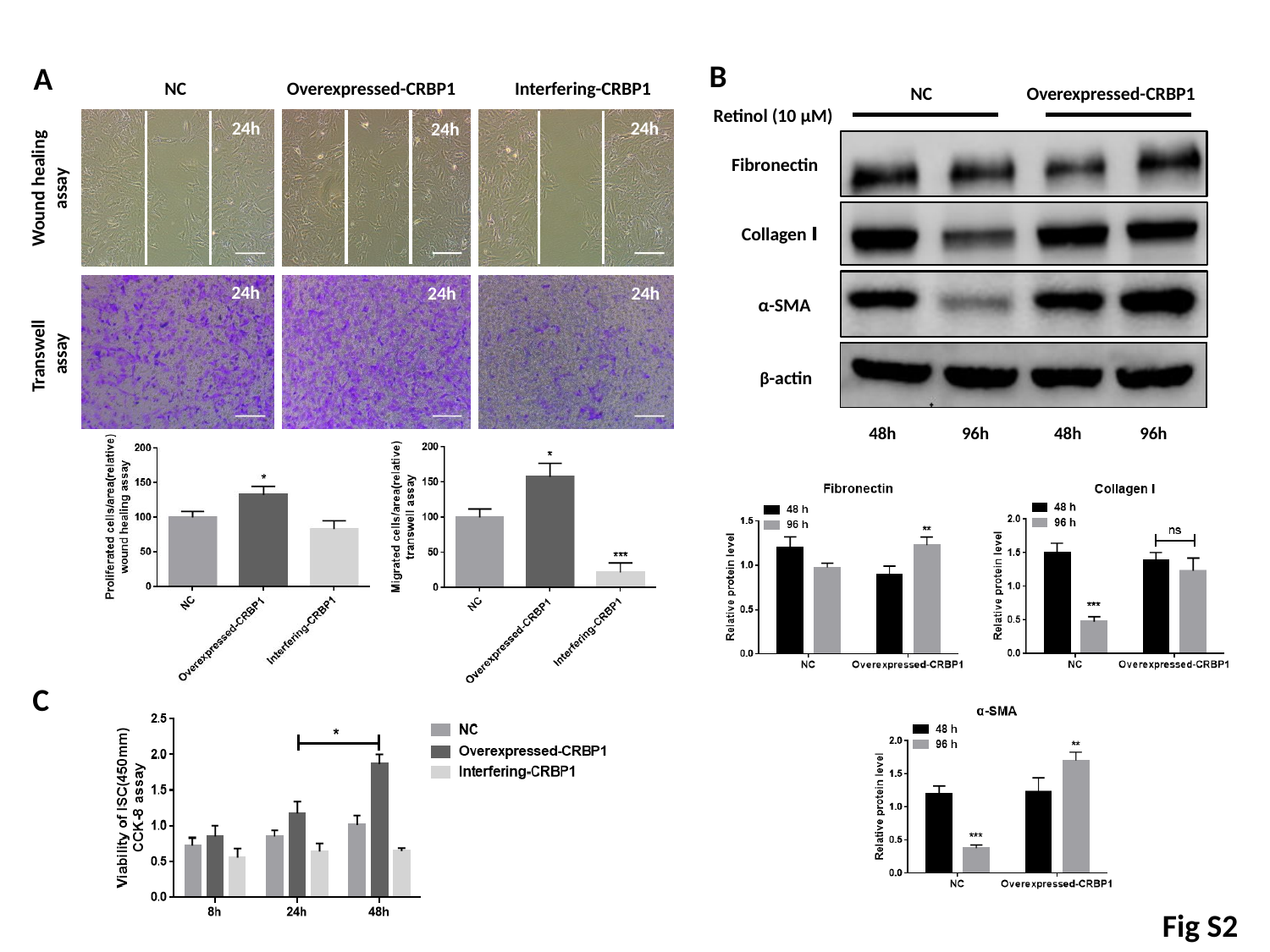

B
Overexpressed-CRBP1
NC
Retinol (10 μM)
Fibronectin
Collagen Ⅰ
α-SMA
β-actin
48h
96h
48h
96h
A
NC
Overexpressed-CRBP1
Interfering-CRBP1
24h
24h
24h
Wound healing
assay
24h
24h
24h
Transwell
assay
C
Fig S2
