# Supplementary material for: Vitamin A deficiency causes islet dysfunction by inducing islet stellate cell activation via cellular retinol binding protein 1
Source: Int J Biol Sci. 2020 Jan 30;16(6):947–56. doi: 10.7150/ijbs.37861 (PMC7053333; doi:10.7150/ijbs.37861)
Supplement: Supplementary file 1 — Supplementary figures and tables. [file ijbsv16p0947s1.zip › Supplementary materials/Table S1.docx]

**Supplementary Table 1, VA levels in serum and pancreatic tissues**

|  | VAS 6W | VAD 6W | VAS 12W | VAD 12W | VADR |
| --- | --- | --- | --- | --- | --- |
| Serum (μmol/L) | 1.56±0.17 | 1.66±0.10 | 1.63±0.14 | 1.63±0.18 | 1.86±0.04 |
| Pancreas (pmol/mg) | 1.66±0.12 | 1.13±0.14^a^ | 1.66±0.20 | 0.61±0.09^a^ | 1.67±0.13 |

^a^values shows a significant effect in post-hoc comparisons vs control group after one-way ANOVA analysis.
