# Supplementary figures and images for: Vitamin A deficiency causes islet dysfunction by inducing islet stellate cell activation via cellular retinol binding protein 1
Source: Int J Biol Sci. 2020 Jan 30;16(6):947–56. doi: 10.7150/ijbs.37861 (PMC7053333; doi:10.7150/ijbs.37861)

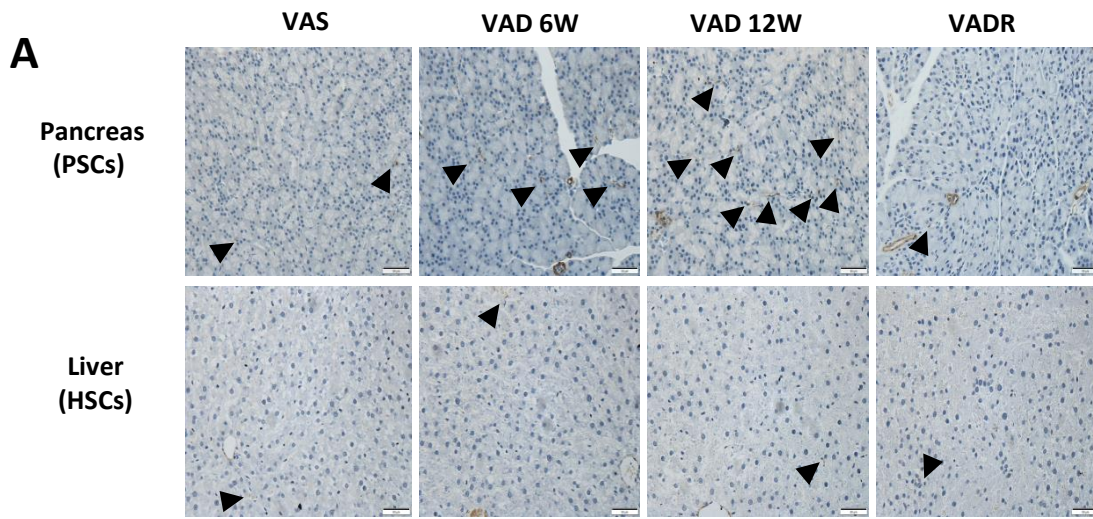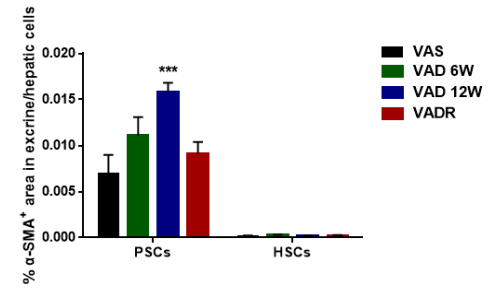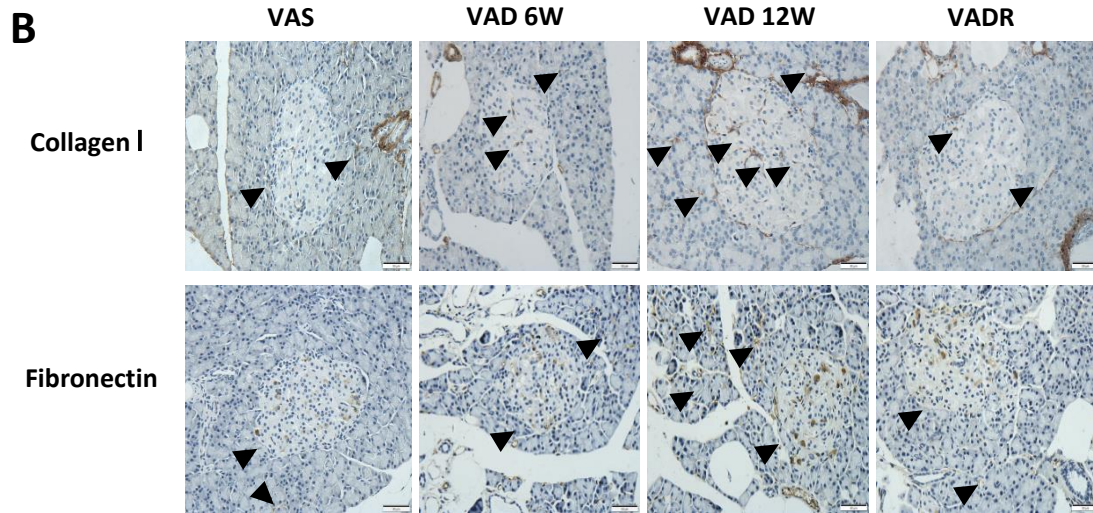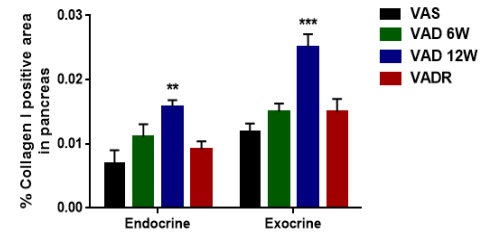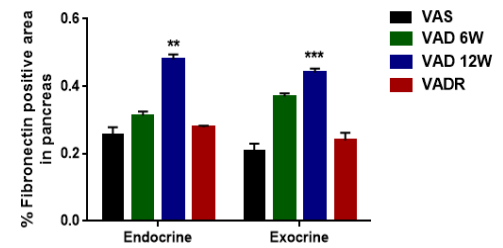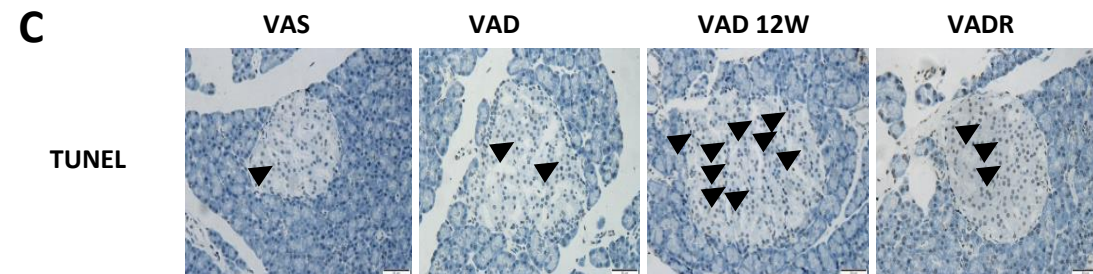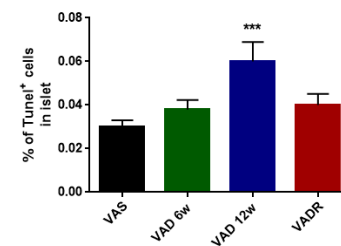

**Fig S1**

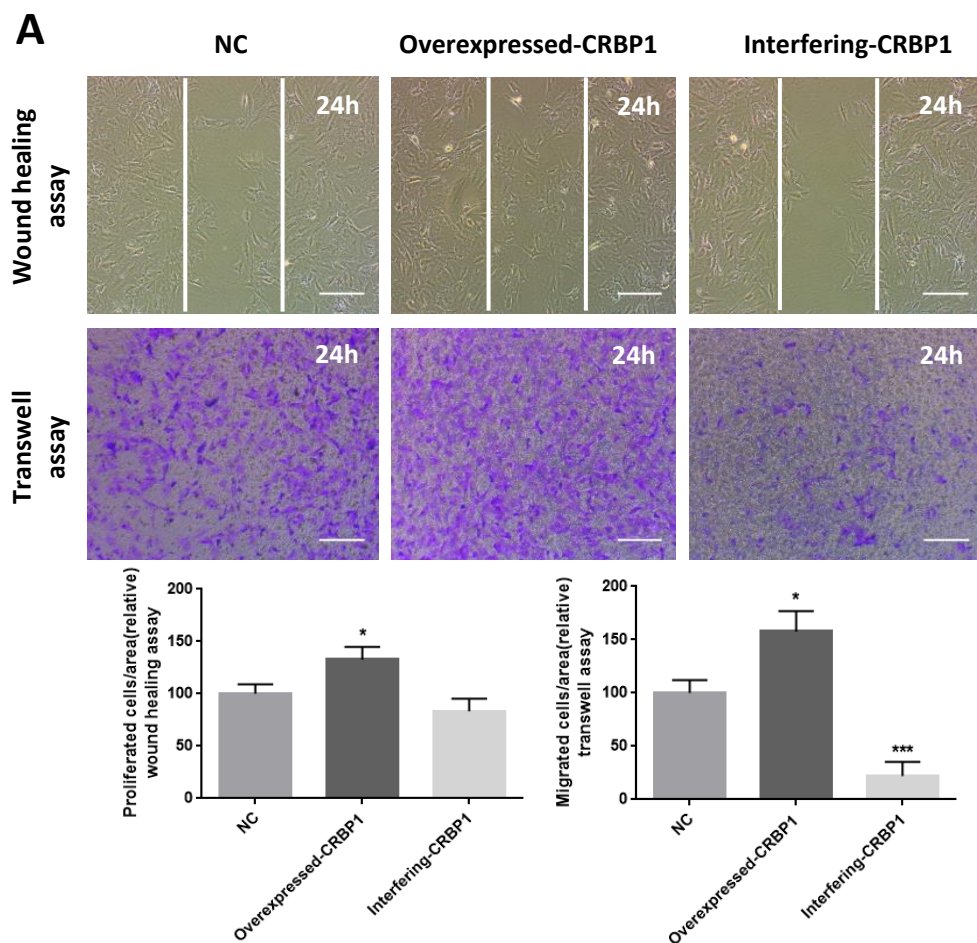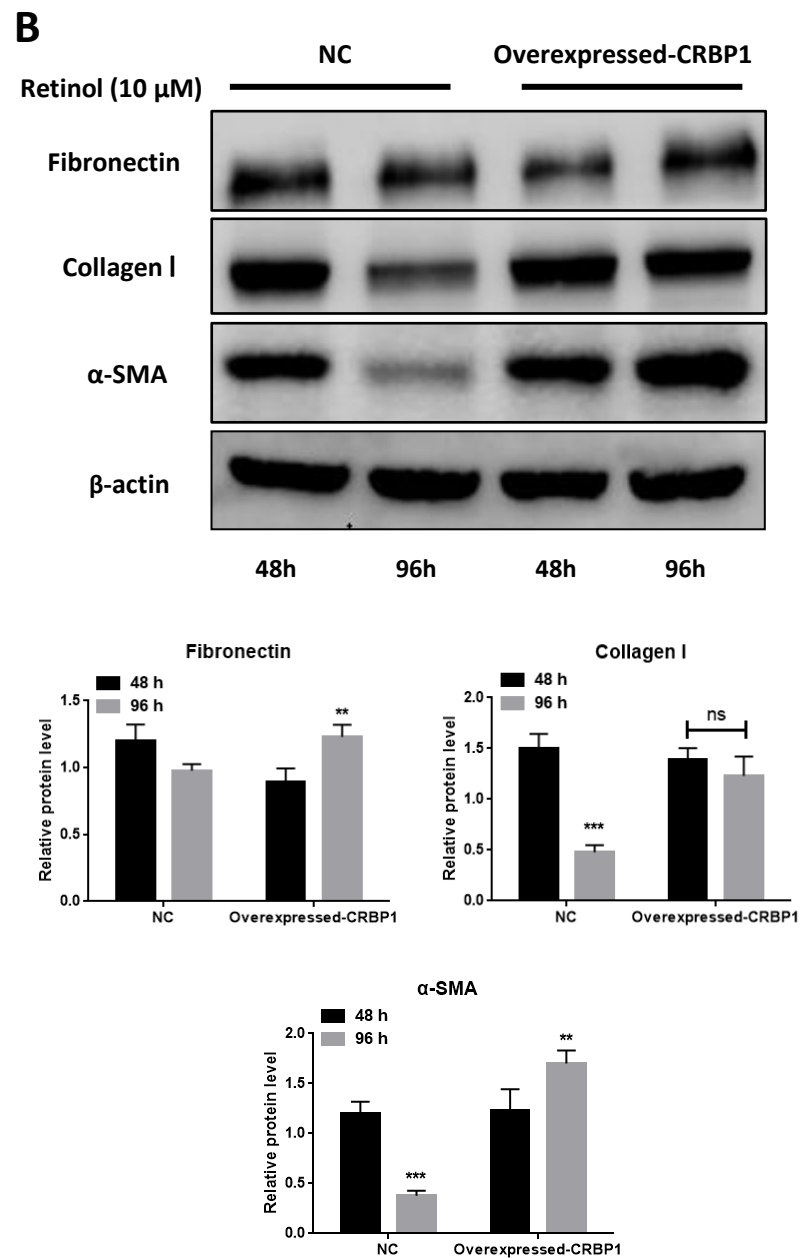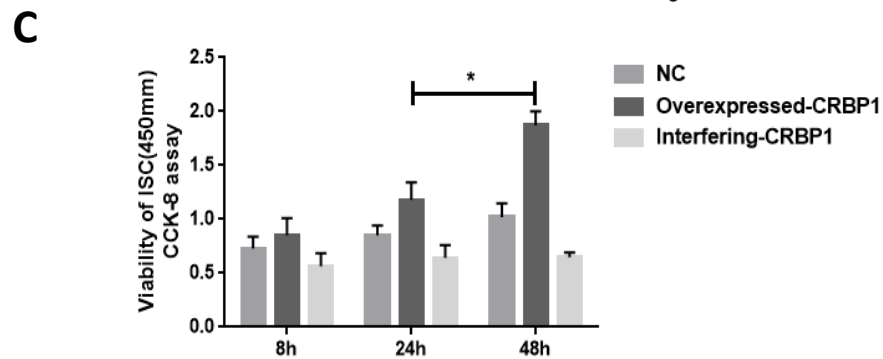

**Fig S2**

Supplement: Supplementary file 1 — Supplementary figures and tables. [file ijbsv16p0947s1.zip › Supplementary materials/Supplementary materials-Figure.pdf]
